# A Fast Room Temperature NH_3_ Sensor Based on an Al/p-Si/Al Structure with Schottky Electrodes

**DOI:** 10.3390/s17081929

**Published:** 2017-08-22

**Authors:** Suwan Zhu, Xiaolong Liu, Jun Zhuang, Li Zhao

**Affiliations:** 1 Collaborative Innovation Center of Advanced Microstructures, State Key Laboratory of Surface Physics and Department of Physics, Fudan University, Shanghai 200433, China; swzhu14@fudan.edu.cn; 2 Shanghai Ultra-Precision Optical Manufacturing Engineering Center and Department of Optical Science and Engineering, Fudan University, Shanghai 200433, China; 15110720002@fudan.edu.cn

**Keywords:** silicon, ammonia, gas sensor, microstructure

## Abstract

In this paper, an electrical-based NH_3_ sensor with an Al/p-Si/Al structure is reported. The p-Si substrate is microstructured by fs-laser irradiation and then etched with 30% alkaline solution. This sensor works well at room temperature with fast response/recovery for NH_3_ gas at 5–100 ppm concentration. However, when the sensor is annealed in N_2_/H_2_ forming gas or short-circuited for Al/Si electrodes, its sensitivity decreases drastically and almost vanishes. Further I-V and FT-IR results show that the two back-to-back Schottky diodes on the device play a key role in its sensing performance.

## 1. Introduction

Ammonia is a frequently-used industrial gas, irritating at high concentration but inconspicuous at lower concentration, that can cause cumulative health effects on human being with continuous exposure. Different types of ammonia sensors have been developed based on different materials in application to specific scenarios [1]. For example, metal-oxide gas sensors typically operate at elevated temperatures independent of humidity and are highly sensitive [2,3]. Magnetic nanofluids are used to develop optical gas sensor that are useful for online monitoring [4]. Conducting polymers like polyaniline (PANI) can be prepared on flexible substrates and shows fine reproducibility through gravure printing [5,6,7]. In recent years, low-dimensional materials have aroused extensive interests, such as carbon-based materials like carbon nanotubes (CNTs) [8,9], graphene oxide (GO) [10] and reduced graphene oxide (rGO) [11], which can be functionalized through different synthesis methods to improve gas selectivity.

As it is the most important material in the semiconductor industry, silicon is often chosen as a substrate material for gas sensors because of its easy integration with silicon-based circuits. But for resistive gas sensing, e.g., for ammonia gas, pristine silicon material shows relatively weak response. In order to improve gas response of silicon material, porous silicon has been given extensive consideration due to its large surface to volume ratio which gives it the ability to react with gases and sense them readily [12,13,14,15,16,17,18]. Generally speaking, higher porosity and larger surface area can improve the sensitivity of the gas sensors, while the response and recovery time are usually longer [16,17]. One of the methods for improving both sensitivity and response time, is to optimize the parameters of porous silicon during the preparation process [18].

In the present work, we fabricate a resistive NH_3_ sensor based on a special microstructured silicon, with the hope of enlarging the sensing area and improving the sensor response. Meanwhile, because such a microstructure is distinct from the porous structure, we expect the sensor will also have a fast response and recovery time. The microstructured substrate is prepared using laser-textured silicon etched by hot a concentrated alkaline solution. Two aluminum electrodes are deposited onto the front of the silicon substrate to form a sensor with an Al/p-Si/Al structure. Room temperature (RT, 25 °C) sensing results indicate that such a silicon sensor with a moderate large surface to volume ratio, achieves not only short response and recovery time at consecutive 5–100 ppm NH_3_, but also an improved response compared to that of the pristine silicon material. To investigate the causes for the improved sensitivity, the sensing properties of the devices whose electrodes are annealed and short-circuited are further studied. Unexpectedly, the results indicate that the improved sensitivity derives from the two Schottky diodes of the sensor, other than the large surface area, which stems from the microstructure.

Although gas sensors based on Schottky barriers have been reported, the electrodes that form the contact play different roles. For sensors based on catalytic metals such as Pd [19,20] or Pt [21], the metal film is a part of the Schottky diode and, at the same time, acts as sensing area. Furthermore, in a similar structure of Al-porous silicon single Schottky junction [22], the author focuses on the interaction of adsorbed molecules with the dangling bonds on the interface. However, in this paper, we compare the different types of contacts and demonstrate that the Schottky contact can enhance sensor response under proper conditions. The relevant research has not been reported.

## 2. Materials and Methods

As a first step, we prepared a special microstructured silicon by using intense femtosecond-laser irradiation [23,24]. Double-polished p-type (100) silicon wafers with resistivity of 1–3 Ω·cm are cleaned by standard RCA process [25] and then mounted on a three-axis translation stage in a stainless-steel chamber with a transparent glass against the stage. The chamber is vacuumized and then backfilled with 70 kPa SF_6_ gas. The wafers are irradiated at normal incidence with a Yb:KGW fs-laser (1 kHz train of 190 fs, 515 nm laser pulses) at a fixed fluence of 8 kJ/m^2^. The laser beam is focused on the sample with a 250-mm focal length lens. A textured area of 10 × 5 mm^2^ is prepared by translating each silicon wafer by a stepper motor in continuous raster scan pattern at a speed of 0.5 mm/s.

**Figure 1:**
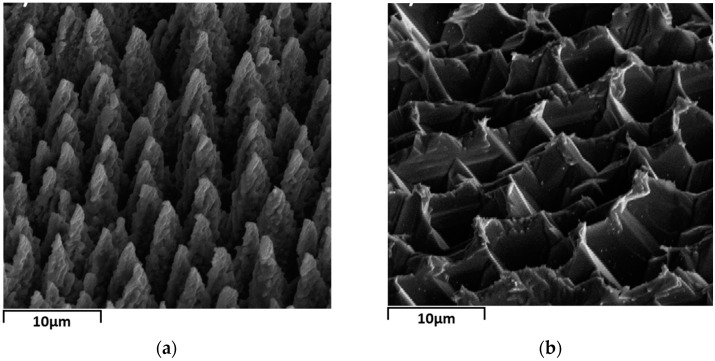
SEM micrographs (viewed at 45°) of laser-textured silicon surface (**a**) before and (**b**) after alkaline etching.

The sample is not ready to use immediately as a sensing material because, firstly, plume is produced in the laser-ablation process [26], in which some clusters are attached to the surface by physisorption and may contribute to poor metal contacts. Secondly, the surface is covered with a thin layer of disordered material [27,28] that serves as a barrier between the conductive bulk and the sensitive surface. Thirdly, the habitual way of storing samples in organic solvents like acetone [29] to prevent oxidation can passivate the surface dangling bonds with unexpected chemical groups, thus changing the gas adsorption properties of the material. Based on these considerations, 30 wt % KOH solution is prepared and then heated in water bath to 80 ± 5 °C. The as-prepared laser-textured silicon is etched by the heated alkaline solution for about 5 s, rinsed in deionized water and then N_2_-dried at RT. The surface morphology of the laser-textured substrate before and after alkaline etching is viewed with a field-emission scanning electron microscope (FE-SEM, ΣIGMA500, Carl Zeiss AG, Oberkochen, Germany) with an accelerating voltage at 5.0 kv. Figure 1 shows SEM micrographs of the microstructured surfaces. After intense femtosecond-laser irradiation, quasi-regular sharp conical spikes are produced with an average height of 10 μm and a spacing of about 5 μm along the translation path, as shown in Figure 1. The conical spikes are etched and corrugated by the alkaline solution, contiguous near the bottom. The disordered layer beneath the surface of the laser-textured silicon has a thickness of hundreds of nanometers only, determined by transmission electron microscopy [28], so the etching process exposes the pristine silicon body. The final microstructure with a depth-diameter ratio of about 1 (10 μm:10 μm) is as shown in Figure 1b. Although the depth-diameter ratio for porous silicon can be changed by different preparation parameters, the typical value of this is usually greater than 10 [15,16,17], which means that our microstructured silicon has a moderately large surface to volume ratio. After the etching process, a pair of aluminum electrodes (3 × 3 mm^2^, 400 nm thickness, for each) is formed by thermal evaporation onto the surface.

**Figure 2:**
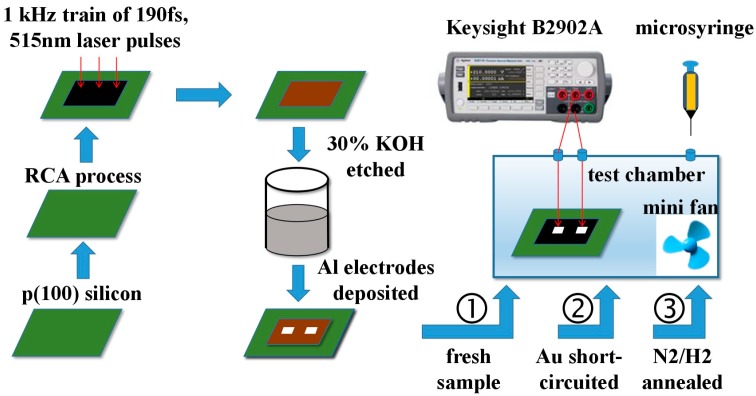
Schematic diagram of sensor fabrication and sensing test system.

To measure the sensor response in NH_3_ gas, a sensing test system is set up. The sensor is placed in a sealed organic glass test chamber in a constant RT/RH laboratory. The test chamber is filled with clean air before sensing measurement. The sensor’s contacts are made an electrical connection with two silver test probes. A stirring mini-fan inside the chamber is used to promote the uniformity of the gas mixture. A predetermined amount of pure gas is injected into the chamber directly by a micro-injector to obtain the desired concentration, which is calculated from the volume ratio of pure NH_3_ to test chamber according to the static volumetric method [30]. Dynamic resistance during sensing measurement is transferred to a PC by using a precision measurement unit (B2902A, Keysight Technologies Inc., Santa Rosa, CA, USA). The schematic diagram of sensor fabrication and testing is shown in Figure 2.

## 3. Results and Discussion

### 3.1. Dynamic NH_3_ Response

**Figure 3:**
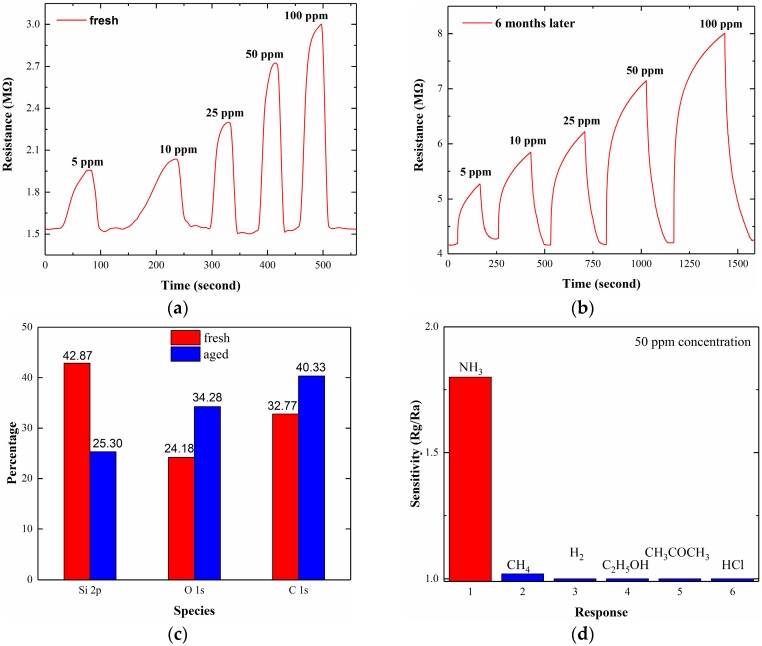
Dynamic response of (**a**) Fresh sample and (**b**) sample stored in air for 6 months versus NH_3_ concentrations ranging from 5 to 100 ppm. (**c**) The proportion of Si 2p, O 1s and C 1s species in the sample before and after ageing. (**d**) Selectivity of the sensor for 50 ppm NH_3_, CH_4_, H_2_, C_2_H_5_OH, CH_3_COCH_3_ and HCl.

The sensing test is carried out at RT with a RH of ~40%. The sensor’s resistance at 0.3 V bias is obtained directly between the two electrodes on the top of the substrate. We choose this bias for two reasons; first, to avoid breakdown of the Schottky electrodes; and second, to reach a relatively weak background current. The details are discussed in the following sections. Figure 3a shows the dynamic response of the freshly prepared sensor versus the concentration of NH_3_ from 5 to 100 ppm. The sensor shows a high sensitivity: an increase in the resistance can be seen even at a concentration as low as 5 ppm, in which a ratio of Rg/Ra (Rg > Ra) is ~1.3, where Ra is the sensor resistance in air and Rg is the dynamic resistance in the targeted gas. Compared with graphene- or CNT-based ammonia sensors where the maximum sensor response to 500 ppm at RT is 30% [10], the response to 100 ppm, shown in Figure 3, is ~100% based on the definition of sensor response (Rg − Ra)/Ra in ref [10]. The response time is defined as the time consumption of a sensor to achieve 90% of the total resistance change in the ambient of the targeted gas, or the recovery time in the ambient of clean air. The response and recovery time of our sensor are less than 25 s and 10 s, respectively, at a typical concentration of 50 ppm. The dynamic curve suggests that the baseline shift is reversible or the recovery appears to be complete, indicating a reversible adsorption of NH_3_ molecules on the silicon’s surface. To check the sensor response for long term stability, the freshly-prepared sample is placed in air for natural ageing. After 6 months, gas response of the aged sample is measured and shown in Figure 3b at concentration from 5 to 100 ppm NH_3_. Compared to the freshly-prepared sample, the air-stored sample exhibits a much more stable response curve. Meanwhile, it can be noticed that the response time and recovery time have been prolonged, which may be attributed to the oxygen and carbon passivation of the silicon surface due to the high surface coverage of these species derived from the air. This view is underpinned by XPS detection in Figure 3c, in which the increase of both O 1s and C 1s species for the aged sample can be found.

Gas selectivity at 50 ppm concentration is also studied in different gases such as NH_3_, CH_4_, H_2_, C_2_H_5_OH, CH_3_COCH_3_ and HCl under the same experimental conditions. As is shown in Figure 3d, only a relatively stronger response of ~1.02 is detected in CH_4_ ambient. The sensor exhibits a much larger response to NH_3_ than to other referenced gases.

The sensor’s resistance increases on exposure to NH_3_ in both Figure 3a,b, exhibiting a typical p-type conductivity where holes are the majority carriers. It is well known that NH_3_ is a reducing agent that can donate electrons to the sensing material [17]. The influence of NH_3_ on mesoporous silicon has been demonstrated by IR-absorption and conductivity variations [31]. Similar to porous silicon, our microstructured silicon belongs to the surface-controlled type in which the effective specific surface area and microstructure characteristics are the main factors determining the gas-sensing properties. However, there is still a lack of consensus for the exact NH_3_-sensing mechanism of the porous silicon. Here we draw on some principles from p-type oxide semiconductors [32], the electrons injected into silicon through the oxidation reaction between NH_3_ and Si-surface decrease the concentration of holes in silicon, which in turn increases the sensor resistance. Although the materials and surface characteristics are not the same, the simplified electron transfer during redox process may explain the resistive changes.

### 3.2. Improved Response with Schottky Electrodes

**Figure 4:**
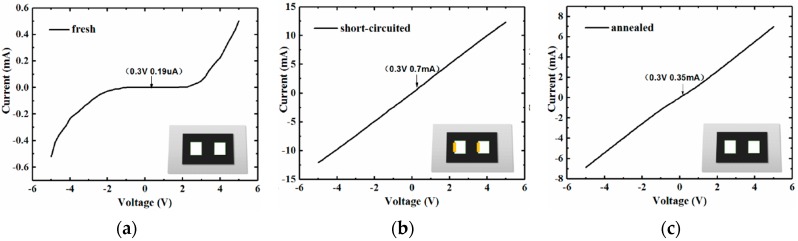
I-V curves of (**a**) freshly prepared, (**b**) short-circuited and (**c**) annealed sensors.

**Figure 5:**
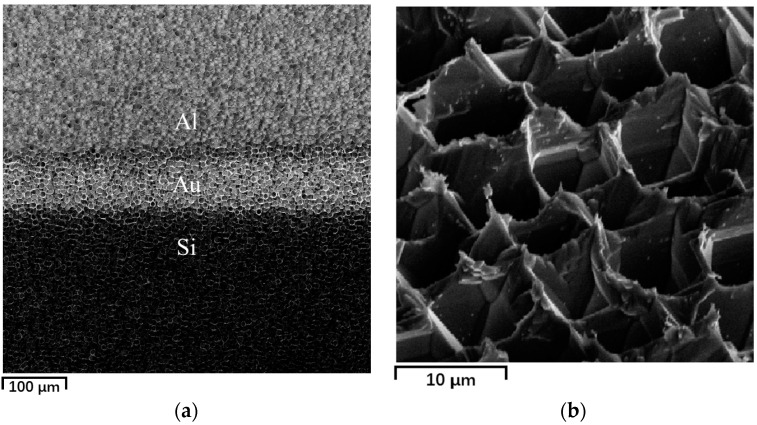
SEM micrographs of the treated sensors. (**a**) Short-circuited electrode (viewed at 0°). (**b**) Surface morphology after annealing (viewed at 45°).

To explore the mechanism of the sensor’s improved response, we have conducted several experiments including an I-V test and an annealing/circuit-shorting sensing test, followed by a FT-IR and XPS test. The I-V curve of the freshly prepared sensor is displayed in Figure 4a, which reveals the existence of two Schottky junctions on the surface of the microstructured silicon in series-opposing conditions. To investigate the influence of the Schottky electrodes on gas response, the rectifying junctions from the Al/p-Si/Al structure are eliminated by two methods. One is to form Si-Al alloy junctions by annealing the device in a forming gas (95% N_2_ and 5% H_2_) at 450 °C for 3 min. The other is to short the Schottky electrodes by depositing a narrow layer of Au (100 nm thickness) at the joint of each Al/p-Si junction using thermal evaporation. I-V characteristics of the treated sensors are displayed in Figure 4b,c, respectively. As can be clearly seen from Figure 4, both treated sensors show that the electrodes have been transformed into Ohmic contacts. Further SEM micrographs display the Al/Au/Si structure and the annealed silicon surface, as shown in Figure 5. In Figure 5a, the presence of a narrow gold layer is designed for eliminating the Al/p-Si junction. In addition, Figure 5b shows that sensor morphology has been mostly retained after annealing at the elevated temperature.

**Figure 6:**
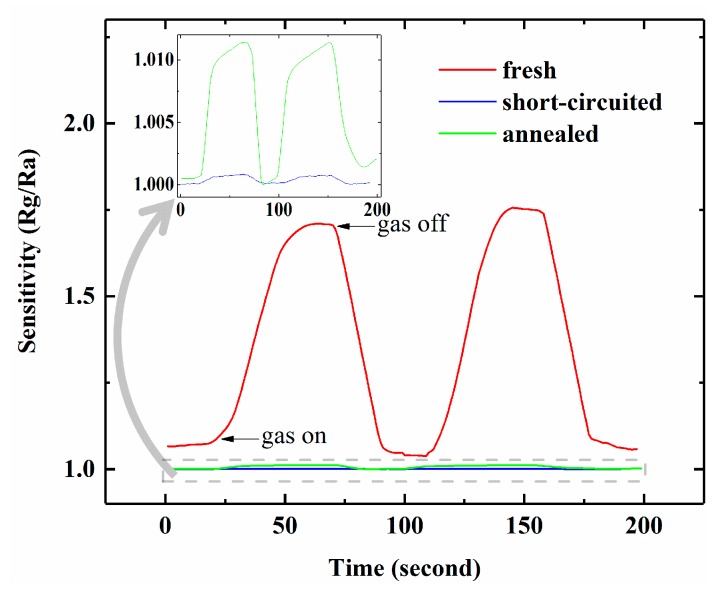
NH_3_ response of freshly prepared, short-circuited and annealed sensors at 50 ppm concentration.

The NH_3_ response of the freshly prepared and treated sensors at 50 ppm concentration are exhibited in Figure 6. Unexpectedly, the gas sensitivity declines drastically to <~1.015 for either treated devices. The inset of Figure 6 suggests that the sensors after treatment still show a very weak response to NH_3_, and this vestigial response mainly results from the semiconducting property of the pristine Si material. Furthernore, the annealed sensitivity is higher than the short-circuited one, this phenomenon is probably due to the incomplete Ohmic annealing process as revealed in Figure 4c.

**Figure 7:**
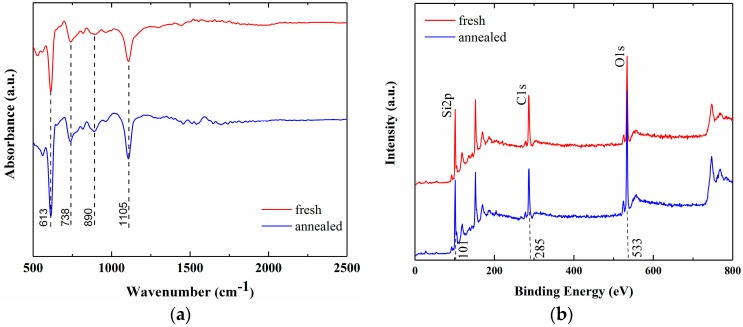
Spectra of (**a**) FTIR and (**b**) XPS obtained from freshly prepared and annealed sample.

To rule out other possible uncertainties raised by N_2_/H_2_ annealing to the material, FT-IR (Bruker Optics ALPHA) and XPS (PHI 5000C ESCA System) spectra are measured to detect the chemical bonds. Figure 7 demonstrates the FT-IR and XPS spectra of the sensors before (red curve) and after (blue curve) annealing treatment. As can be seen in Figure 7a, after gas annealing, no additional chemical component has been introduced to the sensor in the range of 500–2500 cm^−1^ wavenumber that covers the information of Si-Si, Si-O-Si and SiH_x_, etc. [33]. In addition, Figure 7b shows the XPS spectra giving the peaks associated with silicon, carbon and oxygen species. In the fresh sample, the binding energies of Si 2p, C 1s and O 1s are centered at 101, 285 and 533 eV respectively. Moreover, it can be observed that the sample after annealing shows no significant changes for these peaks. Based on these factors, the Al/p-Si Schottky electrodes undoubtedly play a crucial role in accounting for the improved NH_3_ response, instead of the larger surface area stems from our microstructured silicon.

How to comprehend the improved sensitivity for the Schottky device? The variation of the baseline current ΔI_b_ resulting from the presence of NH_3_ gas was reviewed for the three sensors, showing that the values are of the same order of magnitude (~0.3 μA, 0.2 μA and 0.4 μA respectively), indicating that ΔI_b_ is mainly the resistance change of the pristine-Si surface after NH_3_ adsorption. However, the baseline current (current in the air) I_b_ at 0.3 V bias for the three sensors differ enormously, and are ~0.19 μA, 0.7 mA and 0.35 mA respectively, as shown in Figure 4. The gas sensitivity defined as Ra/Rg is positively associated with the value of ΔI_b_/I_b_. Accordingly, when the Schottky junctions are eliminated, I_b_ increases dramatically, thus resulting in a sharp decrease of ΔI_b_/I_b_. This can account for the distinct disparity of the sensitivity between the Schottky device and the Ohmic ones.

### 3.3. Effective Adsorption Sites

**Figure 8:**
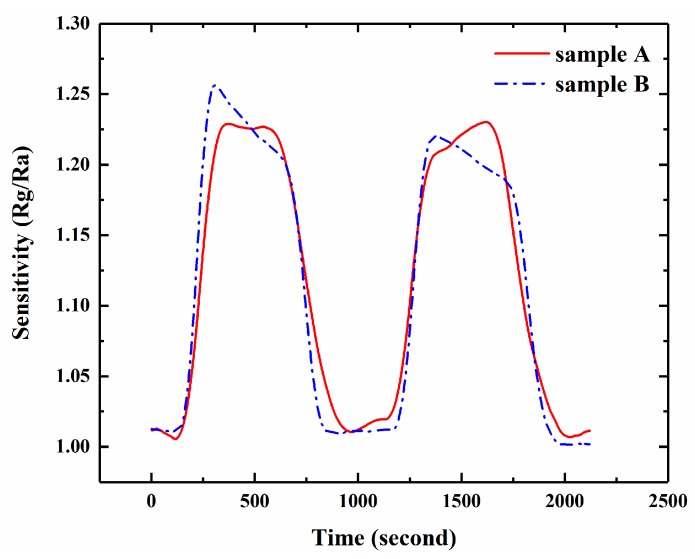
Electrode-coating experiment: NH_3_ response at 5 ppm concentration.

Finally, we also investigate the effective adsorption sites for gas response on the sensor by conducting a contrast experiment. The experiment is carried out with two sensors: one is the freshly prepared device (sample A), and the other is the device with vaseline coating on the surface of the electrodes (sample B). NH_3_ response at 5 ppm concentration for the two samples is shown in Figure 8. and no essential sensing disparity can be found. This result indicates that the effective adsorption sites in the sensor occur only on the interface between the gas and the silicon surface, not on either the Al electrodes or on the Al-Si Schottky junction.

## 4. Conclusions

In summary, an Al/p-Si/Al microstructured NH_3_ sensor is fabricated and studied. The sensor shows not only a moderate sensitivity but also a fast response-recovery rate at RT. I-V curves reveal the existence of two Schottky diodes on the surface of the microstructured silicon. When Schottky junctions are eliminated, the NH_3_ sensitivity of the devices almost vanishes. These results suggest that the NH_3_ sensitivity of our silicon material is improved by the Al/p-Si Schottky diodes connected to p-Si body rather than the high surface area. Furthermore, an electrode-coating experiment reveals the effective adsorption sites for sensor response. Our studies not only enrich the silicon-based NH_3_ sensors for fast response operating at RT, but also present a way of improving the sensor response. In other words, by fabricating adaptive electrical structures and properties to reduce the background signal, one can significantly improve the weak response of other electrical-based sensing materials.
